# Leukemia Cell Lines: In Vitro Models for the Study of Chronic Neutrophilic Leukemia

**DOI:** 10.3390/curroncol28030166

**Published:** 2021-05-10

**Authors:** Hans G. Drexler, Stefan Nagel, Hilmar Quentmeier

**Affiliations:** 1 Faculty of Life Sciences, Technical University of Braunschweig, 38106 Braunschweig, Germany; 2 Department of Human and Animal Cell Lines, Leibniz-Institute DSMZ-German Collection of Microorganisms and Cell Cultures, 38124 Braunschweig, Germany; sna@dsmz.de (S.N.); hqu@dsmz.de (H.Q.)

**Keywords:** cell lines, CNL, leukemia, model

## Abstract

Chronic neutrophilic leukemia (CNL) is a rare myeloproliferative neoplasm that is genetically characterized by the absence of both the Philadelphia chromosome and BCR-ABL1 fusion gene and the high prevalence of mutations in the colony-stimulating factor 3 receptor (CSF3R). Additional disease-modifying mutations have been recognized in CNL samples, portraying a distinct mutational landscape. Despite the growing knowledge base on genomic aberrations, further progress could be gained from the availability of representative models of CNL. To address this gap, we screened a large panel of available leukemia cell lines, followed by a detailed mutational investigation with focus on the CNL-associated candidate driver genes. The sister cell lines CNLBC-1 and MOLM-20 were derived from a patient with CNL and carry CNL-typical molecular hallmarks, namely mutations in several genes, such as CSF3R, ASXL1, EZH2, NRAS, and SETBP1. The use of these validated and comprehensively characterized models will benefit the understanding of the pathobiology of CNL and help inform therapeutic strategies.

Chronic neutrophilic leukemia (CNL) is a distinct but rare myeloproliferative neoplasm that is BCR-ABL1 negative. CNL is diagnosed on the basis of neoplastic expansion of granulocytic cells and is also included in the WHO classification of hematological malignancies [1,2]. Epidemiology, demographics, histopathology, and clinical and laboratory diagnostic features of CNL have been reviewed in detail elsewhere [3,4]. Historically, any insight into the pathogenetic basis of CNL has been limited as traditional cytogenetics has been normal in the majority of patients at diagnosis [5,6,7].

CSF3R is the receptor for colony-stimulating factor 3 (previously referred to as granulocyte colony-stimulating factor) which is known to play a key role in the growth and differentiation of granulocytes [8]. Mutations in CSF3R were found in the majority of CNL patients and were hence thought to be germane to the biology of CNL [1,9]. This discovery was validated in several cases series of CNL [6,10,11,12,13,14].

The CSF3R mutations fall into two classes: the so-called truncation mutations (a premature truncation of the cytoplasmic tail of the CSF3R) and membrane proximal mutations (point mutations in the extracellular domain), most commonly T618I [3]. The mutation T618I confers ligand independence and leads to constitutive activation of JAK/STAT signaling [15,16]. Mice transplanted with CSF3R T618I-expressing hematopoietic cells developed a fatal myeloid neoplasm [17].

These data suggest that high-frequency oncogenic mutations in the CSF3R are a defining molecular abnormality of CNL and thus clearly represent a major diagnostic criterion [1,3]. Recent informative genomic data showed that CSF3R mutations do not occur alone. The most common concurrent mutations occur in the genes ASXL1, SETBP1, SRSF2, TET2, and EZH2 (Figure 1A). Thus, a more sophisticated genomic profile of CNL suggests mutational cooperativity.

The survival of CNL patients is dismal and the therapeutic options are limited and do not exhibit proven disease-modifying benefits [3]. This unsatisfactory situation prompted investigation of alternative approaches. The inhibition of kinase signaling downstream of mutated CSF3R was considered a feasible molecularly targeted therapy [3,19]. However, the rarity of the disease has been a serious challenge. Though data are evolving in this small field, speedier improvement of therapeutic interventions has been hampered by the limited body of knowledge of genetic and cellular underpinnings which, in part, also owes to the lack of representative in vitro cell models. Hence, it appears essential to establish a leukemia cell line model which replicates the in vivo situation [20,21,22]. Furthermore, it is preferable to use cell lines with particular genomic aberrations as proxies for biological features prevailing in the in vivo space.

Previously, a pair of cell lines was established from a patient with CNL, albeit at different time points during disease progression [22,23]. These cell lines were designated as CNLBC-1 and MOLM-20. We conducted a comprehensive evaluation of the cell lines. In particular, we charted the genomic landscape of CNLBC-1 and MOLM-20 in our molecular workup of a specially assembled panel of leukemia–lymphoma cell lines [24]. The salient features of these two CNL cell lines are summarized in Table 1 and shown in part in Figure 1. The two cell lines are clonally related since they have identical DNA fingerprints and carry the same cytogenetic and genetic characteristics. Additionally, the phenotypical details are shared by the two cell lines.

Most importantly, both cell lines carry the telltale CSF3R mutation, specifically the T618I variant (Figure 1B). In addition, a further four genes are mutated: ASXL1 (Y591*), EZH2 (I146T), NRAS (G12D), and SETBP1 (D868N) (Table 1). Among the ten most common mutations occurring in CNL patients, five (the top three and two more) were also found in CNLBC-1 and MOLM-20 (Figure 1A), attesting to the genetic fidelity and thus the suitability of these cell lines to represent CNL models. This high level of concordance in the genetic landscape between primary samples and cell line MOLM-20 was not seen in the other 99 leukemia–lymphoma cell lines that had been characterized in our previous thorough and systemic genomic screen [24].

In summary, a major impediment to further investigation of CNL is the lack of informative and faithful models that allow functional interrogation of driver genes and the impact of the acquisition of additional mutations. To address this knowledge gap, we have highlighted here the existence of such a valuable CNL model in the form of two sister cell lines and have undertaken a global characterization of these cells. The presence of typical molecular hallmarks indicates the use of these cell lines as vital preclinical models in the analysis of CNL pathogenesis and in the search for therapeutics.

Sister cell lines CNLBC-1 and MOLM-20 have been established from a patient with CNL. Both cell lines are characterized by a distinct mutation landscape which corresponds to that of primary CNL samples, in particular, they carry the CNL-typical mutations CSF3R, ASXL1, and SETBP1. The cell line MOLM-20 is available from the public cell line repository DSMZ (www.dsmz.de, accessed on 1 January 2021).

## Figures and Tables

**Figure 1:**
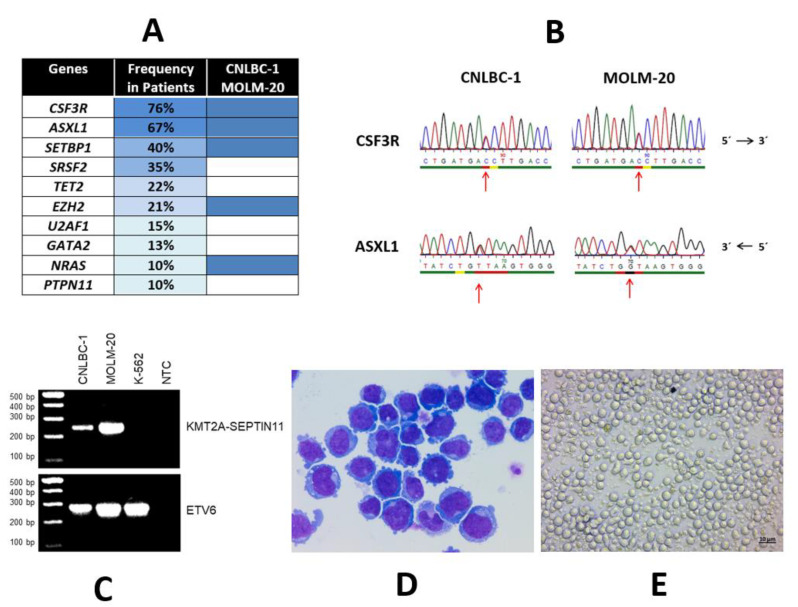
Mutational landscape of CNL and characteristics of CNL cell lines CNLBC-1 and MOLM-20. (**A**) Mutational landscape in CNL. Left column: mutated genes; middle column: frequency of mutations in primary cases (the mutation frequency of the listed genes was calculated from several case series [6,10,11,12,13,14]; right column: mutations present in CNL cell lines CNLBC-1 and MOLM-20. While CSF3R mutations are associated with CNL, *ASXL1* is frequently mutated in various myeloid malignancies, including myelodysplastic syndromes, chronic myelomonocytic leukemia, and acute myeloid leukemia. SETBP1 mutations are found in several myelodysplastic/myeloproliferative entities. (**B**) Characteristic genomic alterations in CNLBC-1 and MOLM-20. Shown here: point mutations in CSF3R (T618I, COSM4169901/COSM1737962) and ASXL1(Y591*, COSM1681609) detected with Sanger sequencing in both CNLBC-1 and MOLM-20. (**C**) Cytogenetic alterations of CNLBC-1 and MOLM-20 which also carry a t(4;11)(q21;q23) leading here to the fusion gene KMT2A-SEPTIN11 [18]. Shown here: reverse transcription polymerase chain reaction analysis of fusion gene KMT2A-SEPTIN11 in CNLBC-1 and MOLM-20; additionally, cell line K-562 (derived from CML) was used as negative control; ETV6 served as control for cDNA used; NTC, no template control. This t(4;11)(q21;q23) should not be confused with the cytogenetically identical t(4;11)(q21;q23) which molecularly leads to the fusion KMT2A-AFF1 (formerly known as MLL-MLLT2) and which occurs more often in acute lymphoblastic leukemia. The uncropped PCR agarose blot is shown in Figures S1. (**D**) Morphology of CNLBC-1 cells. Shown here: May–Grünwald–Giemsa-stained cells after cytospin centrifugation on glass slide. (**E**) Cell culture of CNLBC-1 cells. Shown here: cells grown in suspension culture in 24-well plate. Cell line CNLBC-1 was obtained from Dr. I. Sakai, Ehime, Japan. Cell line MOLM-20 was obtained from Dr. Y. Matsuo, Okayama, Japan.

**Table 1:** Key features of CNL cell lines CNLBC-1 and MOLM-20: clinical, culture, cytogenetic, genomic, and immunophenotypic data.

Cell Lines	CNLBC-1	MOLM-20
Patient	63-year-old woman ^1^	64-year-old woman ^1^
Disease diagnosis	CNL	CNL
Disease status	in transformation/at blast crisis	at relapse (patient died shortly thereafter)
Specimen site	peripheral blood	peripheral blood
Year established	April 2002	March 2003
Authenication of cell line	yes (by cytogenetics, fusion gene)	yes (by STR profiling)
Culture	RPMI 1640 medium + FBS at standard conditions	RPMI 1640 medium + FBS at standard conditions
Doubling time	36 h	70 h
Viral status	EBV−	EBV−, HBV−, HCV−, HIV−, HTLV-I/II−
Karyotype	49, XX, +X, +8, +21, t(4;11)(q21;q23)	49(47–50)<2n>XXX, +X, +8, +21, t(4;11)(q21.1;q23)
Fusion gene	KMT2A-SEPTIN11 (previously MLL-SEPT11/FLJ10849)	KMT2A-SEPTIN11 (previously MLL-SEPT11/FLJ10849)
Gene mutations	ASXL1 Y591*, CSF3R T618I, EZH2 I146T, NRAS G12D, SETBP1 D868N (EZH2 mutation is homozygous, all other mutations are heterozygous)	ASXL1 Y591*, CSF3R T618I, EZH2 I146T, NRAS G12D, SETBP1 D868N (EZH2 mutation is homozygous, all other mutations are heterozygous)
Immunoprofile	T/NK: CD2−, CD3−, CD4+, CD5−, CD7−, CD10−, CD56+, CD57− B: CD10−, CD19−, CD20−, CD22−, CD79a− MyMon: CD13+, CD14+, CD33+, MPO+ EryMeg: CD41−, CD61− other: CD34+, HLA-DR−, TdT−	T/NK: CD3−, CD4+, CD7−, CD56+ B: CD10−, CD19−, CD20−, smIg− MyMon: CD13+, CD14(+), CD15+, CD33+, CD68+, MPO+ EryMeg: CD41− other: CD34+, CD45+, HLA-DR−, TdT−
Publication	ref. [23]	refs. [22,24]

^1^ The patient was treated with hydroxyurea which induced a partial hematological response. A leukemic transformation occurred 8 months after diagnosis. She died of refractory leukemia 16 months after initial diagnosis [23]. Abbreviations: B, B cell; CD, cluster of differentiation (immunoprofile); CNL, chronic neutrophilic leukemia; EBV, Epstein–Barr virus; EryMeg, erythrocytic–megakaryocytic; FBS, fetal bovine serum; HBV, hepatitis B virus; HCV, hepatitis C virus; HIV, human immunodeficiency virus; HTLV, human T cell leukemia virus; MPO, myeloperoxidase; MyMono, myeloid/monocytic; NK, natural killer; smIg, surface membrane immunoglobulin; STR, short tandem repeat; T, T cell, TdT, terminal deoxynucleotidyl transferase.

## Data Availability

Data can be found at www.dsmz.de and at ENA under the accession number PRJEB30297 and PRJEB30312.

## Supplementary Materials

The following are available online at https://www.mdpi.com/article/10.3390/curroncol28030166/s1, Figure S1: the whole blot (uncropped blots) for Figure 1C.
