# Supplementary material for: Leukemia Cell Lines: In Vitro Models for the Study of Chronic Neutrophilic Leukemia
Source: Curr Oncol. 2021 May 10;28(3):1790–4. doi: 10.3390/curroncol28030166 (PMC8161829; doi:10.3390/curroncol28030166)
Supplement: Supplementary file 1 [file curroncol-28-00166-s001.zip › curroncol-1162396 supplementary materials.pdf]

Current Oncology

Brief Report

Leukemia Cell Lines: In Vitro Models for the Study of Chronic Neutrophilic Leukemia

Hans G. Drexler, Stefan Nagel and Hilmar Quentmeier

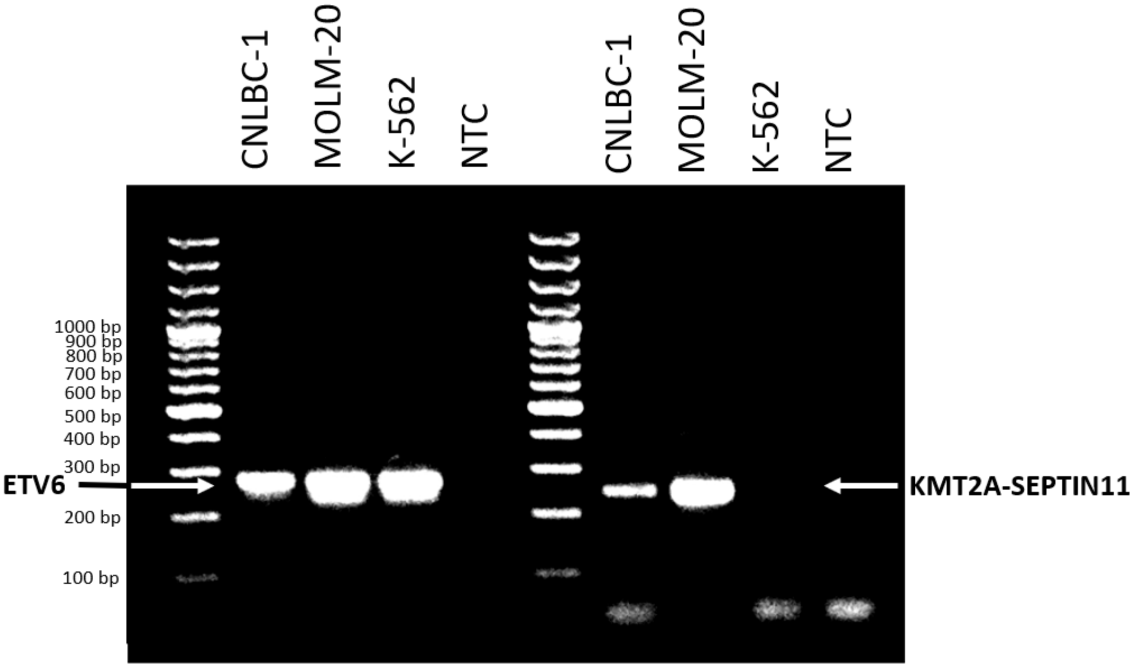

Figure S1. the whole blot (uncropped blots) for Figure 1C.
